# Gene expression profiling in NOD mice reveals that B cells are highly educated by the pancreatic environment during autoimmune diabetes

**DOI:** 10.1007/s00125-022-05839-7

**Published:** 2022-12-12

**Authors:** Joanne Boldison, Jessica R. Hopkinson, Joanne Davies, James A. Pearson, Pia Leete, Sarah Richardson, Noel G. Morgan, F. Susan Wong

**Affiliations:** 1 Department of Clinical and Biomedical Sciences, University of Exeter, Exeter, UK; 2 Division of Infection and Immunity, Cardiff University School of Medicine, Cardiff, UK

**Keywords:** Autoimmunity, B cells, Pancreatic islets, TLR7, Type 1 diabetes

## Abstract

**Aims/hypothesis:**

B cells play an important role in driving the development of type 1 diabetes; however, it remains unclear how they contribute to local beta cell destruction during disease progression. Here, we use gene expression profiling of B cell subsets identified in inflamed pancreatic tissue to explore their primary functional role during the progression of autoimmune diabetes.

**Methods:**

Transcriptional profiling was performed on FACS-sorted B cell subsets isolated from pancreatic islets and the pancreatic lymph nodes of NOD mice.

**Results:**

B cells are highly modified by the inflamed pancreatic tissue and can be distinguished by their transcriptional profile from those in the lymph nodes. We identified both a discrete and a core shared gene expression profile in islet CD19^+^CD138^–^ and CD19^+^CD138^+^ B cell subsets, the latter of which is known to have enriched autoreactivity during diabetes development. On localisation to pancreatic islets, compared with CD138^–^ B cells, CD138^+^ B cells overexpress genes associated with adhesion molecules and growth factors. Their shared signature consists of gene expression changes related to the differentiation of antibody-secreting cells and gene regulatory networks associated with IFN signalling pathways, proinflammatory cytokines and Toll-like receptor (TLR) activation. Finally, abundant TLR7 expression was detected in islet B cells and was enhanced specifically in CD138^+^ B cells.

**Conclusions/interpretation:**

Our study provides a detailed transcriptional analysis of islet B cells. Specific gene signatures and interaction networks have been identified that point towards a functional role for B cells in driving autoimmune diabetes.

**Graphical abstract:**

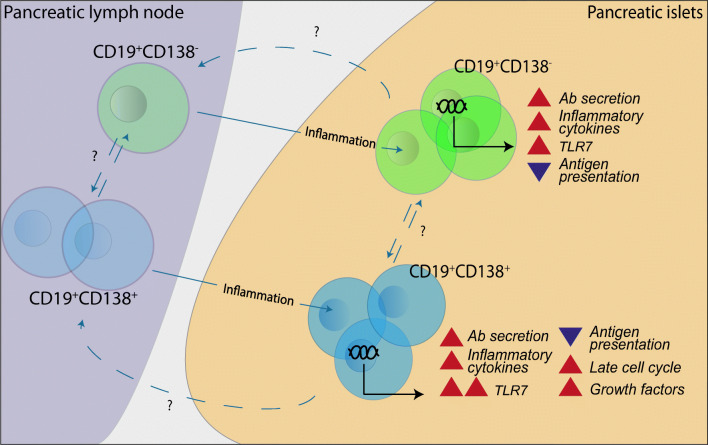

**Supplementary Information:**

The online version of this article (10.1007/s00125-022-05839-7) contains peer-reviewed but unedited supplementary material..

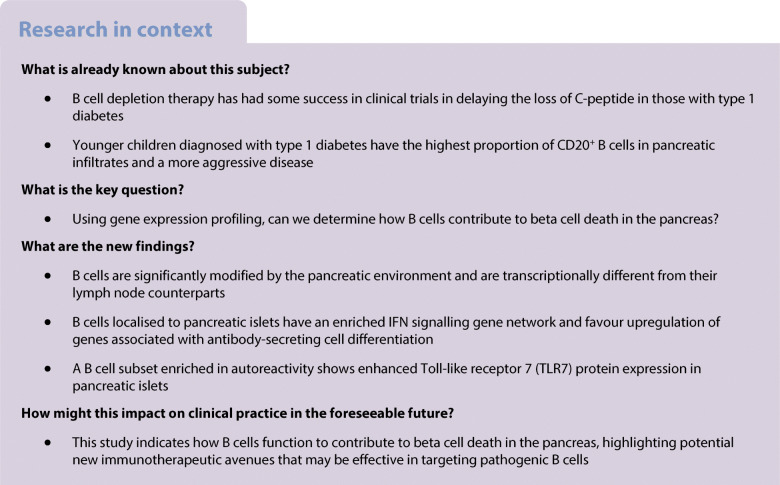



## Introduction

Type 1 diabetes is an organ-specific autoimmune disease characterised by immune-mediated beta cell destruction, resulting in insulin deficiency. Among current immune-targeting therapies, B cell depletion therapy (rituximab) has successfully temporarily delayed the loss of C-peptide in type 1 diabetes [1] and this success has also been mirrored in numerous animal studies [2, 3]. Immunohistological analysis has revealed the presence of pancreatic CD20^+^ B cells in individuals with type 1 diabetes [4, 5] and their number correlates with age at diagnosis. Younger children (<13 years at disease onset) have the greatest proportion of infiltrating CD20^+^ cells and more aggressive disease [6], highlighting the need for further interrogation of the role of B cells in immune-mediated destruction of pancreatic beta cells.

In the NOD mouse model, B1 B cells that are present in the pancreas early in the disease process are important in the initiation of type 1 diabetes [7, 8], whereas established islet B cells have a more follicular phenotype [9]. During established insulitis, B cells become CD5 negative and upregulate CD138 (syndecan-1) [10, 11], a common plasma cell marker [12].

We have described distinct pancreatic populations of B cells in NOD mice during a stage of aggressive insulitis that are distinguished by the expression of CD138, IgD and CD19 [11, 13] (see Electronic supplementary material [ESM] Table 1). These subsets included a population of B cells that expressed CD138 at an intermediate level and importantly were enriched with insulin-specific autoreactive B cells [11]. A small subset of proliferating B cells that resemble a murine plasmablast phenotype [14], characterised by high levels of expression of CD138 and CD44 and lower levels of expression of CD19, were noted [13]. We also identified a CD138^+^ subset that showed reduced CD19 and IgD expression [11]. Here, we use comprehensive transcriptional analyses of these distinct B cell subsets to explore their primary functional role at the tissue level during the development of autoimmune diabetes.

## Methods

### Mice

NOD/Caj mice, originally from Yale University, were bred and maintained in specific pathogen-free isolators or scantainers at Cardiff University, UK. All animals received water and food ad libitum and were housed in a 12 h dark/light cycle. The animal experiments were conducted in accordance with the UK Animals (Scientific Procedures) Act 1986 and associated guidelines. Female 16- to 20-week-old NOD mice were chosen at random and processed in groups for gene array experiments. Experiments were performed unblinded.

### Tissue preparation

Pancreatic lymph nodes (PLNs) were disrupted and pancreases were inflated as previously described [11, 13]. See ESM Methods for further details.

### Flow cytometry and fluorescent activated cell sorting

Cells were stained as previously described [11, 13]. See ESM Methods for further details of the antibodies used. Cell suspensions were either acquired on an LSRFortessa (BD Biosciences) and analysed using FlowJo v10.1 software (Tree Star, Ashland, OR) or sorted on a FACSAria III (BD Biosciences).

### Immunofluorescence

Pancreatic tissues were frozen in optimal cutting temperature (OCT) medium, sectioned at 7–10 μm thickness and stained as previously described [13]. See ESM Methods for further details.

### RNA isolation

RNA was isolated from cells using the Qiagen RNeasy Micro Kit, according to the manufacturer’s instructions. See ESM Methods for further details.

### Clariom S array

RNA amplification was achieved using low-cycle PCR followed by linear amplification using T7 in vitro transcription technology. See ESM Methods for further details.

### Gene array analyses

CEL files generated from Affymetrix Clariom S mouse arrays were imported into the Bioconductor package oligo v1.56.0 [15] or Transcriptome Analysis Console (TAC) software version 4.0 (ThermoFisher). First, DNA microarray analyses were performed in R v4.0.3 and normalised using the Robust Multichip Average (RMA) algorithm [16]. Differential expression analysis was performed using the linear models for microarray data (limma) package v3.48.1 [17]. Linear models were determined for each transcript cluster and global variance was calculated using an empirical Bayes approach [18]. A moderated *t* statistic was computed for each transcript cluster with the resulting *p* values corrected using the Benjamini–Hochberg method to control for the false discovery rate (FDR). See ESM Methods for more details.

### Quantitative PCR

Samples were prepared for quantitative PCR (qPCR) using a EPMotion P5073 liquid handling robot (Eppendorf) and amplified in duplicate alongside a housekeeping gene on a ViiA7 real-time PCR system (ThermoFisher). Normalisation of samples was performed by dividing the value of the gene of interest by the value of the housekeeping gene (*Gapdh*) (ΔC_t_) and a mean ΔC_t_ is presented.

### Statistical analysis

Statistical analyses were performed in R software (https://www.r-project.org/index.html) or GraphPad Prism software (GraphPad Software, San Diego, CA). Genes were discarded if differential expression was not significant (adjusted *p* value <0.05; Benjamini–Hochberg correction for multiple testing). Other statistical tests used are provided in the figure legends.

## Results

### Abundance of B cell subsets in the pancreas during autoimmune diabetes development

**Fig. 1:**
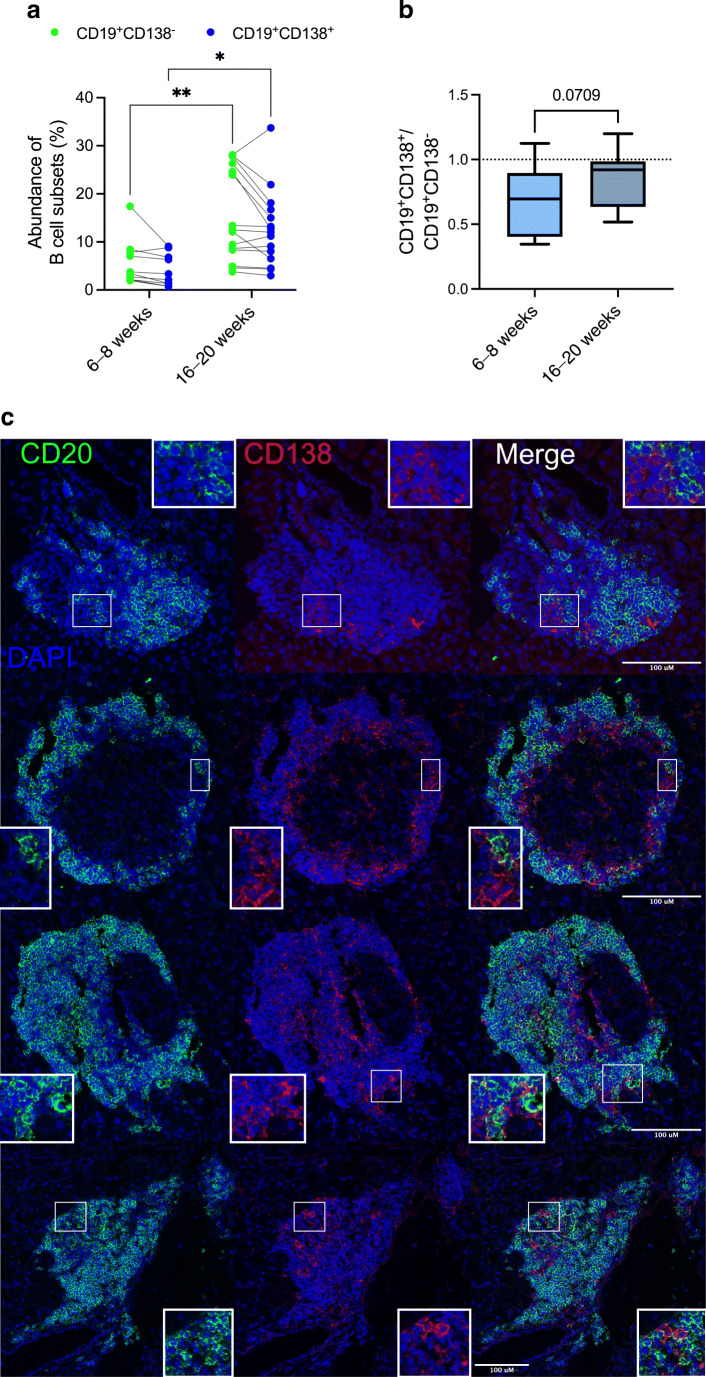
Abundance of B cell subsets in the pancreas during autoimmune diabetes development. (**a**, **b**) Pancreatic islet cells from either single or groups of female NOD mice aged 16–20 weeks were analysed by flow cytometric gating on CD19^+^CD138^–^ (green) and CD19^+^CD138^+^ (blue) B cells. (**a**) Overall abundance of both CD19^+^ B cell subsets compared with that in younger NOD mice assessed previously [13]. Each dot represents either one mouse or pooled mice. *p<0.05, **p<0.01 by two-way ANOVA with Bonferroni’s multiple comparison test. (**b**) Ratio of CD19^+^CD138^+^ to CD19^+^CD138^–^ B cells in younger and older NOD mice. *p*=0.0709 by Mann–Whitney *U* test. Data are from five independent experiments. Data represents mean ± SEM. CD19^+^ B cells were gated on live singlets CD3^–^CD11b^–^CD11c^–^ cells. (**c**) Representative images of pancreatic sections from female NOD mice (blood glucose >13.9 mmol/l) stained with anti-CD20 (green), anti-CD138 (red) antibodies and nuclear DAPI counterstain. Scale bars, 100 μm. Images represent pancreatic islets or immune cell clusters from three individual mice

We have previously described B cell subsets localised in pancreatic islets, differentiated according to the expression of CD138, among which a CD19^+^CD138^+^ population (intermediate expression levels) is enriched in autoreactive B cells [11]. We investigated the abundance of CD19^+^CD138^–^ and CD19^+^CD138^+^ islet B cells in NOD mice during diabetes development (Fig. 1). Compared with the abundance of pancreatic CD19^+^CD138^–^ (mean ± SEM 7.1 ± 1.09%) and CD19^+^CD138^+^ (mean ± SEM 3.6 ± 2.7%) B cells in younger NOD mice (6–8 weeks) [13], there was a significant increase in both CD19^+^CD138^–^ (*p*<0.01) and CD19^+^CD138^+^ (*p*<0.05) B cells in 16- to 20-week-old NOD mice (Fig. 1a) and a shift towards a 1:1 ratio as islet inflammation progressed (*p*=0.07) (Fig. 1b). At diabetes onset, we identified both CD138^–^ and CD138^+^ B cells in the remaining insulin-containing islets and in immune cell clusters (Fig. 1c), using CD20 as a B cell marker. CD138^+^ cells, with no CD20^+^ expression, were also evident (Fig. 1c), corroborating our previous phenotyping by flow cytometry [11]. Overall, both CD19^+^CD138^–^ and CD19^+^CD138^+^ B cells were prominent in the pancreatic islet immune infiltrates of NOD mice.

### CD19^+^CD138^–^ and CD19^+^CD138^+^ B cells have similar transcriptional profiles

Previously, in NZB/W mice, splenic CD19^+^CD138^+^ mature B cells were described at an early intermediary antibody-secreting cell (ASC) stage [19]. We now show that NOD splenic CD19^+^CD138^+^ B cells also display a mature follicular B cell phenotype (ESM Fig. 1a–c), in common with islet B cells, during established insulitis (IgD^+^IgM^low^) [9].

**Fig. 2:**
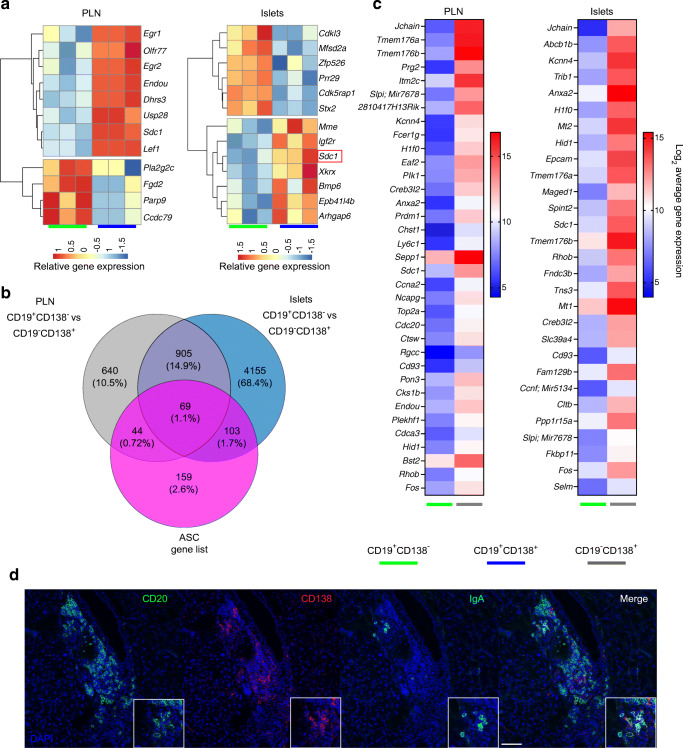
CD19^–^CD138^+^ pancreatic immune cells are enriched with fully differentiated plasma cells. (**a**–**c**) PLNs and pancreatic islets from groups of female NOD mice aged 16–20 weeks were isolated (*n*=8) and CD19^+^CD138^–^ (green), CD19^+^CD138^+^ (blue) and CD19^–^CD138^+^ (grey) cells were FACS sorted for RNA isolation and gene array analysis. (**a**) Unsupervised hierarchical clustering heatmaps of the DEGs (uncorrected *p*<0.001) from PLNs and pancreatic islets comparing CD19^+^CD138^–^ and CD19^+^CD138^+^ B cells. Three individual sorted populations were arrayed for each B cell subset; each column represents the relative gene expression from one experimental sample. The red box highlights the *Sdc1* (syndecan-1 or CD138) gene expressed at an FDR<0.05. (**b**) Venn diagram showing overlaps between an ASC gene list and genes that were significantly (FDR<0.05, more than a twofold change) upregulated or downregulated in CD19^+^CD138^–^ and CD19^–^CD138^+^ cells in both PLN and pancreatic tissue. (**c**) Heatmaps showing the top ASC-associated genes differentially overexpressed in CD19^–^CD138^+^ cells compared with CD19^+^CD138^–^ cells in PLNs and pancreatic islets. Average (mean) gene expression is shown from three individual samples. (**d**) Representative images of pancreatic sections from female NOD mice (blood glucose >13.9mmol/l) stained with anti-CD20 (green), anti-CD138 (red) and anti-IgA (cyan) antibodies and nuclear DAPI counterstain. Scale bar, 100 μm. Images show an immune cell cluster and are representative of three individual mice

We determined the transcriptional profile of the different B cell subsets localised in the pancreas by gene expression array. B cell populations (CD19^+^CD138^–^, CD19^+^CD138^+^, CD19^–^CD138^+^) were purified by FACS (ESM Fig. 1d) from pancreatic islets and the PLNs of pooled 16- to 20-week-old female NOD mice followed by isolation of RNA. Differentially expressed genes (DEGs) with more than a twofold change with an FDR<0.05 were compared between CD19^+^CD138^–^ and CD19^+^CD138^+^ B cells from PLNs and pancreatic islets (Fig. 2). *Sdc1* (encoding CD138) was the only DEG that was upregulated (FDR<0.05) (Fig. 2a) in the CD19^+^CD138^+^ subset, in both PLNs and islets. The heatmap in Fig. 2a demonstrates DEGs with an uncorrected *p* value of <0.001. The upregulated genes in both tissues were compared with data in the Immunological Genome Project (ImmGen) database [20]. CD19^+^CD138^+^ B cells were associated with more mature ImmGen B cell subsets, including memory and plasmablast-like B cells (ESM Fig. 1e). An ASC signature gene, *Endou* [21], was also upregulated in these cells.

### Both innate lymphocytes and fully differentiated plasma cells are enriched in the CD19^–^CD138^+^ cell subset in pancreatic islets

Previously, we suggested that CD19^–^CD138^+^ cells located within the pancreas are a heterogenous mix of B cells differentiating into ASCs [13], owing to the expression of CD138. However, because of the absence of CD19 and the observation that other cell types can express CD138 [22, 23], we wanted to ascertain if the CD19^–^CD138^+^ cell subset expresses ASC- or B cell-associated genes. In PLNs, CD19^–^CD138^+^ B cells were compared with CD19^+^CD138^–^ B cells in the gene array dataset, identifying a significant number of DEGs between these populations (1658 genes; FDR<0.05, more than a twofold change) (ESM Table 2). The top 20 upregulated genes (by fold change) in the CD19^–^CD138^+^ subset were clustered (ESM Fig. 2a), revealing that some of the highly upregulated genes (e.g. *Il7r*, *Cd7*, *Klrb1b*) are abundantly expressed by innate lymphocytes [24, 25]. These findings were replicated in the B cells from the pancreatic islets (ESM Fig. 2b, ESM Table 3). *Jchain* (expressed in plasma cells [20]) was also highly upregulated in CD19^–^CD138^+^ cells (ESM Fig. 2a, ESM Tables 2 and 3). Other DEGs, including *Tmem176a* and *Tmem176b*, are expressed both in plasma cells [26] and in innate lymphoid cells [27]. Upregulated genes in CD19^–^CD138^+^ cells from both tissues were compared with ASC signature genes [21], with shared genes found in both tissues (Fig. 2b). Overexpressed genes included *Prdm1* (B lymphocyte-induced maturation protein 1 [BLIMP-1]) and *Xbp1* (X-box binding protein 1 [XBP-1]) and are essential for plasma cell differentiation [28, 29]. The top plasma cell-related genes (Fig. 2c) were upregulated by more than sevenfold (FDR<0.05). As the *Jchain* gene encodes the protein component of IgA, we examined if IgA^+^ cells are present in the pancreatic tissue of NOD mice. Occasional groups of IgA^+^ cells, mostly CD138^+^, were seen in immune cell clusters (Fig. 2d). Thus, the CD19^–^CD138^+^ subset identified in both PLN and pancreatic tissue in NOD mice contains cells with a more differentiated plasma cell phenotype and a population of innate-like lymphocytes.

### B cell populations are significantly modified by the inflamed pancreatic environment

**Fig. 3:**
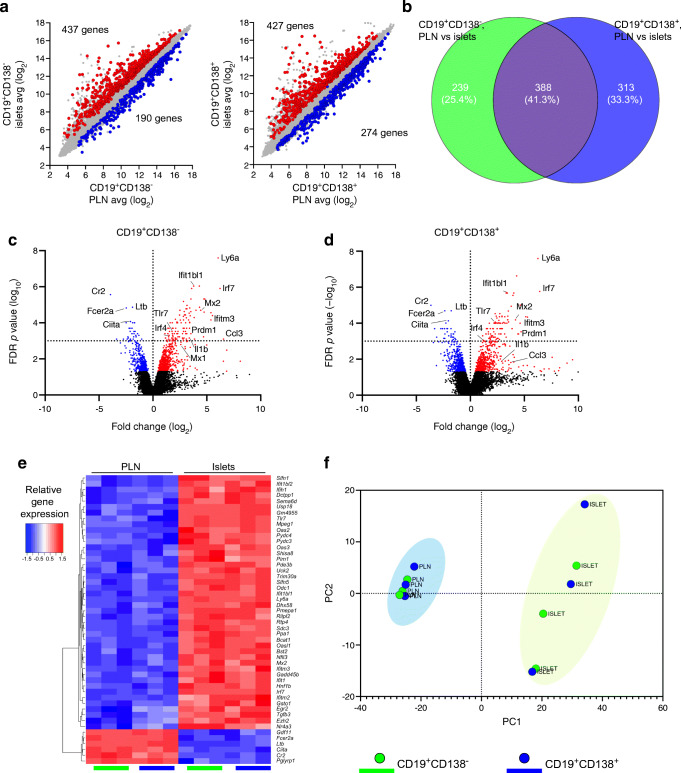
B cell populations are significantly modified by the inflamed pancreatic environment. PLNs and pancreatic islets from groups of female NOD mice aged 16–20 weeks (*n*=8) were isolated and CD19^+^CD138^–^ (green) and CD19^+^CD138^+^ (blue) cells were FACS sorted for RNA isolation and gene array analysis. (**a**) Scatterplots showing upregulated (red) and downregulated (blue) DEGs in the CD19^+^CD138^–^ and CD19^+^CD138^+^ B cell subsets between the PLNs and the pancreatic islets. (**b**) Venn diagrams showing core shared genes and differences between both B cell gene sets on location from PLNs to pancreatic islets. (**c**, **d**) Volcano plots for (**c**) CD19^+^CD138^–^ and (**d**) CD19^+^CD138^+^showing the –log_10_ FDR *p* value (*p*<0.01 indicated above the dashed line) on the *y*-axis and log_2_ fold change on the *x*-axis and annotated genes of interest. Upregulated DEGs between PLNs and pancreatic islets are shown in red and downregulated DEGs are shown in blue (FDR<0.05). (**e**) Hierarchical clustering heatmap showing the top 50 most significant DEGs between PLNs and pancreatic islets for both B cell subsets (FDR<0.05, more than a twofold change). (**f**) Gene clustering of the DEGs (FDR<0.05, more than a twofold change) shown in a principal component analysis plot. Avg, average; PC, principal component

As CD19^+^ B cells represent the more abundant population [13] (vs CD19^-^CD138^+^ cells), we focused on the CD19^+^ B cells in pancreatic islets, investigating the effects of the inflamed environment comparing the B cell subsets with those in the PLNs (Fig. 3). In the CD19^+^CD138^–^ B cell subset, we identified 437 upregulated and 190 downregulated DEGs (Fig. 3a). Similarly, in the CD19^+^CD138^+^ B cell subset, 427 DEGs were upregulated and 274 DEGs were downregulated (Fig. 3a) (FDR<0.05, more than a twofold change). While both B cell subsets from pancreatic islets and PLNs shared DEGs (388 genes, 41.3%, ESM Table 4 [Tab 3]), we identified a substantial number of DEGs that distinguished these two subsets (Fig. 3b, ESM Table 4 [Tab 4, 5]), with individual upregulated (red) and downregulated (blue) genes highlighted in volcano plots (Fig. 3c,d). Hierarchical clustering of the top 50 shared DEGs (Fig. 3e) in both subsets demonstrated that the majority of DEGs were upregulated. Finally, principal component analysis, measuring the transcriptional distance between the subsets in both tissues, revealed two discrete clusters. This confirmed the distinction between the populations found in PLNs and pancreatic islets (Fig. 3f) but highlighted the close relationships between the different CD19^+^ B cell subsets located within the respective tissues, particularly in the PLNs. Taken together these data indicate that both B cell subsets are heavily influenced by their environment yet are transcriptionally similar when resident in the islets but significantly different from their counterparts in PLNs.

### B cells localised in the pancreas are enriched in genes associated with antibody-secreting cells

**Fig. 4:**
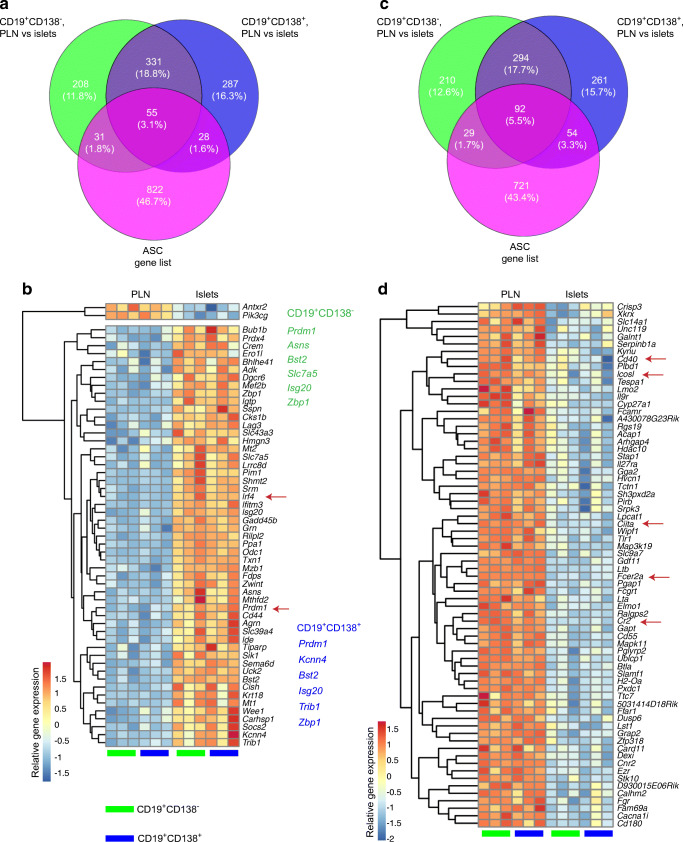
B cells localised in the pancreas are enriched in genes associated with ASCs. DEGs (FDR<0.05, more than a twofold change) in both B cell subsets from the PLN vs pancreatic islet comparison were assessed against published ASC genes, both overexpressed and repressed in the differentiation pathway. (**a**, **c**) Venn diagrams illustrating overlap between genes that are significantly upregulated (**a**) and downregulated (**c**) between the PLN and the pancreatic islets in each cell subset and the ASC gene list. (**b**, **d**) Hierarchical clustering heatmaps showing shared genes in pancreatic islets (compared with PLNs) in both B cell subsets associated with ASCs. (**b**) Genes known to be overexpressed in ASCs (FDR<0.05) corresponding to the B cell subsets. ASC signature genes associated with CD19^+^CD138^−^ and CD19^+^CD138^+^ B cells subsets are listed in green and blue text, respectively; alterations in expression of these genes was highly significant (FDR<0.01). (**d**) Genes known to be repressed in ASCs corresponding to the B cell subsets. Red arrows highlight key factors in B cell differentiation and function. Each column represents the relative gene expression from one experimental sample

We noted that the key genes regulating plasma cell differentiation, *Prdm1* and *Irf4* [30, 31], were upregulated in both islet B cell subsets. Therefore, we compared the gene sets from both B cell populations with genes associated with the ASC differentiation pathway, either as activated or repressed targets [21, 32] (Fig. 4).

Activated genes in the ASC list were compared with DEGs in both gene sets and, surprisingly, both subsets had a modest number of genes in common with genes activated in the ASC pathway (Fig. 4a), with the majority of the shared DEGs being activated (FDR<0.05, more than a twofold change) (Fig. 4b). Additionally, *Ly6a* (stem cell antigen-1 [Sca-1]), a surface antigen used for plasma cell gating strategies in mice [33, 34], was highly upregulated (Fig. 3c,d). Genes that were significantly altered in expression (FDR<0.01) and are part of an ASC gene signature [21] were identified in both CD19^+^CD138^–^ (green) and CD19^+^CD138^+^ (blue) B cells (Fig. 4). Activation of the genes *Ly6a* and *Prdm1* in both subsets in pancreatic islets compared with PLNs was confirmed by qPCR (ESM Fig. 3).

We next assessed the repressed genes identified in our subsets, previously identified as downregulated in ASC [21, 32]. Again, some genes were shared by the populations, while others were exclusive to one or the other (FDR<0.05, more than a twofold change) (Fig. 4c). Of the 92 shared genes, 23 were induced, including several IFN-related genes (listed in ESM Table 5 [Tab 6]). However, key genes involved in antigen presentation and B cell activation, including *Ciita* and *Cd40*, were repressed on localisation of the B cells to pancreatic islets (arrows, Fig. 4d).

We next studied whether CD138^+^ B cells had more mature ASC-like changes on translocation to islets by interrogating the datasets for patterns in the levels of expression of ASC-related genes or for exclusive genes in either subset. We show each B cell gene set that was upregulated and associated with ASCs (ESM Fig. 4). Key genes (*Prdm1* and *Irf4*) were induced to a similar extent (vs their respective PLN subsets). A selection of genes unique to each cell subset was also identified. Genes selectively expressed in CD19^+^CD138^–^ B cells included *Alcam* (activated leukocyte cell adhesion molecule) and *Pycr1* (CD19^+^CD138^+^, FDR>0.1) (ESM Fig. 4), whereas CD19^+^CD138^+^ B cells selectively expressed *Tns3* and *Epcam* (CD19^+^CD138^–^, FDR>0.1). Furthermore, in the CD19^+^CD138^+^ gene set, *Cd9* expression was increased compared with the CD19^+^CD138^–^ gene set (FDR<0.05, more than a twofold change [ESM Table 5, Tab 7]). Similarly, among the repressed genes, *Bcl6*, *Il4ra* and *Ms4a1* (CD20) were restricted to the CD19^+^CD138^+^ subset (ESM Table 5 [Tab 8]). Several genes were significantly changed in the CD19^+^CD138^–^ cells, but by more than twofold. ESM Table 5 (Tab 7, 8) shows all differentially expressed ASC-related genes with an FDR<0.05, irrespective of the extent of the fold change. Of note, *Pax5*, a gene directly repressed by *Prmd1*, was significantly downregulated by >1.5-fold in both subsets. These data show that CD138^+^ B cells have substantially more genes repressed on islet translocation than CD138^–^ B cells, but they do not show expression of a single group of genes characteristic of a more mature ASC cell. Overall, these data suggest that, on arrival in the inflamed environment in pancreatic islets, both B cell subsets favour the induction of key ASC-associated genes.

### Identifying gene expression differences in CD19^+^CD138^+^ B cells in pancreatic islets

**Fig. 5:**
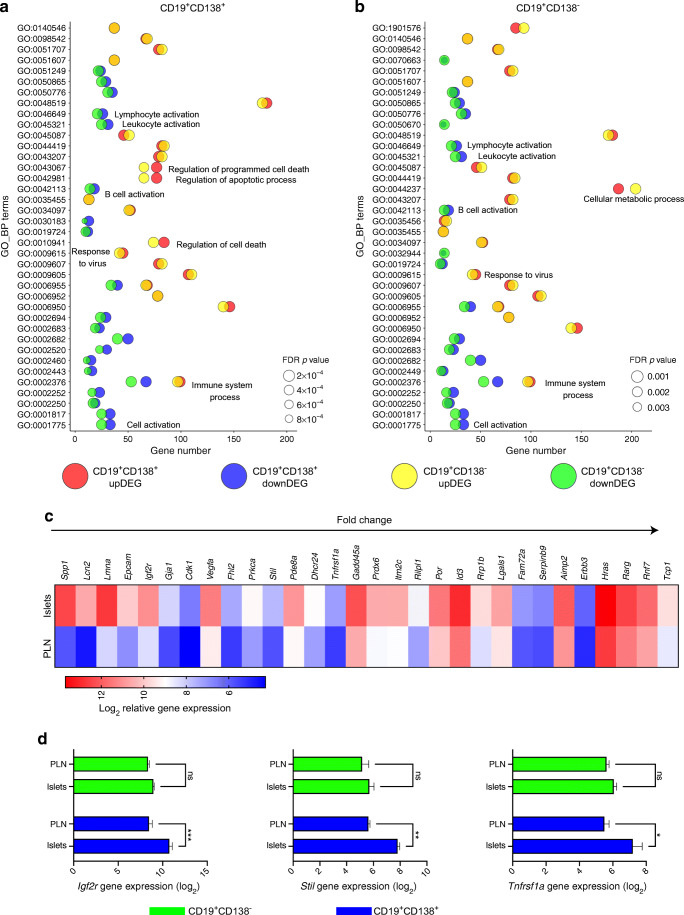
CD19^+^CD138^+^ B cells in the pancreas overexpress growth factor and late cell cycle genes. Genes either up- or downregulated in each gene set (FDR<0.05, more than a twofold change) were used to test for over-represented biological processes using the PANTHER classification system. (**a**, **b**) Bubble plots showing the top 20 over-represented GO terms in each B cell subset. GO terms are plotted against the related gene number identified in either B cell subset. The FDR *p* value is depicted by the size of the bubble. (**a**) GO terms enriched in the CD19^+^CD138^+^ B cell gene set; (**b**) GO terms enriched in the CD19^+^CD138^–^ B cell gene set. (**c**) Heatmap showing average (mean) expression of upregulated DEGs (FDR<0.05, more than a twofold change) in the CD19^+^CD138^+^ B cell gene set (PLNs and pancreatic islets) identified by enriched GO terms related to ‘cell death’ and the ‘apoptotic process’. (**d**) Mean ± SEM gene expression of *Igf2r*, *Stil* and *Tnfrsf1a* in B cell subsets from both PLNs and pancreatic islets. *p<0.05, **p<0.01 and ***p<0.001 from two-way ANOVA with Bonferroni’s multiple comparison test. downDEG, downregulated DEG; GO_BP, GO_biological processes; ns, not significant; upDEG, upregulated DEG

Another goal of our gene profiling experiments was to identify any additional transcriptional changes in the CD19^+^CD138^+^ B cell subset. We therefore extended our investigation using gene ontology (GO) analysis, employing the PANTHER classification system [35] for an ‘in-depth’ analysis of the DEGs in both B cell subsets (Fig. 5). Genes either up or downregulated in each set (FDR<0.05, more than a twofold change) were tested for over-represented biological processes. The bubble plot in Fig. 5a shows the most significant biological processes from DEGs either upregulated (top 20, red) or downregulated (top 20, blue) in CD19^+^CD138^+^ B cells. Gene numbers are plotted against GO terms, while FDR *p* values are indicated by the bubble size. GO terms in the double-positive B cells were compared with gene numbers and corrected *p* values in the upregulated (yellow) and downregulated (green) DEGs in CD19^+^CD138^–^ B cells. Figure 5b shows a bubble plot of the top GO terms for CD19^+^CD138^–^ B cells, with gene numbers and corrected *p* values compared with the double-positive B cells. A full description of GO terms is provided in ESM Fig. 5.

Although many of the top GO terms overlapped, we observed some important differences. The CD19^+^CD138^–^ B cell subset was enriched in upregulated genes associated with metabolic processes. In the CD19^+^CD138^+^ B cells, more B cell activation genes were downregulated, but ‘programmed cell death’ or the ‘apoptotic process’ genes were upregulated. Therefore, we studied the genes associated with these processes and selected 30 genes that were significantly expressed in CD19^+^CD138^+^ cells (compared with CD19^+^CD138^–^ cells; FDR>0.05) (Fig. 5c). However, genes such as *Spp1* and *Lmna* gave a corrected *p* value <0.08 in the CD19^+^CD138^–^ gene set (data not shown). Therefore, we eliminated genes (Fig. 5c) that were expressed in single-positive B cells and had a *p*<0.05 (not corrected), identifying eight genes (*Igf2r*, *Stil*, *Tnfrsf1a*, *Fam72a*, *Serpinb9*, *Erbb3*, *Rnf7* and *Tcp1*) expressed exclusively in CD19^+^CD138^+^ B cells. The top three DEGs expressed only in CD19^+^CD138^+^ B cells are presented in Fig. 5d.

Taken together, these data suggest that CD19^+^CD138^+^ B cells have a subtle but clear difference in their gene profile compared with CD19^+^CD138^–^ B cells on trafficking to the pancreas. On arrival in the vicinity of islets, double-positive B cells appear to overexpress genes associated with adhesion, growth factors and later stages of the cell cycle.

### CD19^+^ B cell subsets acquire an innate immune signature on translocation to pancreatic islets

**Fig. 6:**
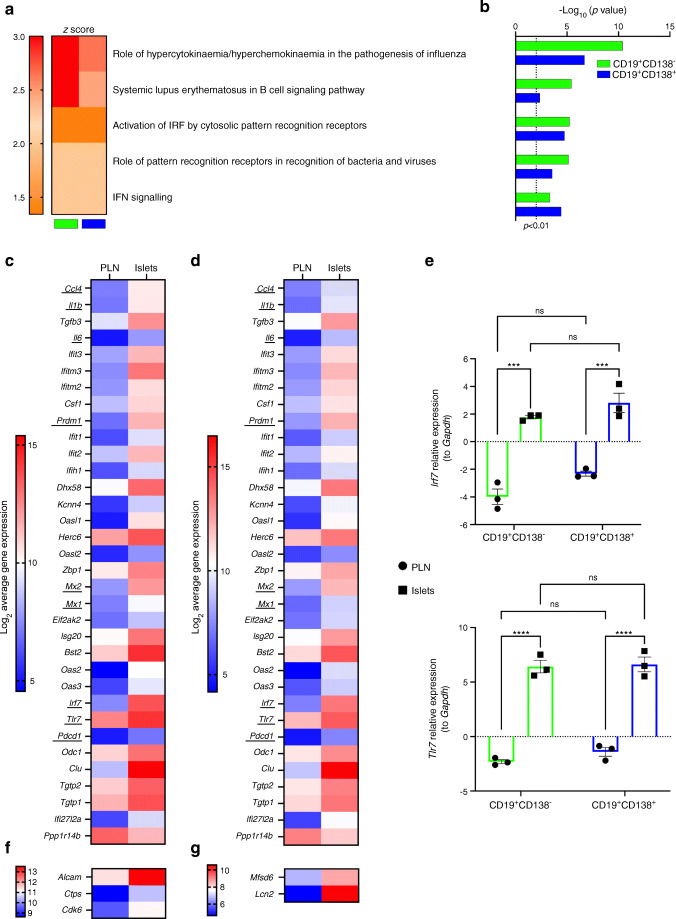
CD19^+^ B cell subsets acquire an innate immune signature on location to pancreatic islets. Enriched gene canonical pathways and networks in B cell subsets (CD19^+^CD138^–^, green; CD19^+^CD138^+^, blue) in pancreatic islets using IPA software (FDR<0.05, 1.5-fold change). (**a**, **b**) Canonical pathways in B cell subsets displaying significant activation denoted by (**a**) *z* scores (orange to red) and (**b**) the corresponding corrected *p* values (dotted line represents *p*<0.01). (**c**, **d**) Expression profiles of innate or IFN-related signature genes shared between subsets: (**c**) CD19^+^CD138^–^, (**d**) CD19^+^CD138^+^. Underlining highlights genes of interest. (**e**) qPCR of *Irf7* and *Tlr7* genes in B cell subsets located in PLNs and pancreatic islets. Expression was related to the housekeeping gene *Gapdh*. ***p<0.001 from two-way ANOVA with Bonferroni’s multiple comparison test. (**f**, **g**) Expression profile of innate or IFN-related signature genes not shared between subsets: (**f**) CD19^+^CD138^–^ (**g**) CD19^+^CD138^+^. ns, not significant

We next explored enriched gene regulatory networks in B cells localised in the pancreas using ingenuity pathway analysis (IPA) (Fig. 6). This provided the top five canonical pathways that displayed significant increases in activation (corrected *p*<0.01). The activation status of each canonical pathway, for each B cell subset, is denoted by the *z* score (orange = activated) in Fig. 6a, with corresponding *p* values for each pathway in Fig. 6b. Activated pathways indicated that IFN-induced gene expression is enriched in B cells. We used the activated IPA pathways and GO analysis to identify signature genes upregulated by CD19^+^CD138^–^ (Fig. 6c) and CD19^+^CD138^+^ (Fig. 6d) B cells after translocation. Most genes were significantly upregulated in both B cell subsets, including cytokines encoded by *Il6* (IL-6), *Il1b* (IL-1β) and *Ccl4* (macrophage inflammatory protein 1β [MIP-1β]), transcription factors encoded by *Irf7* and *Prdm1* and key antiviral proteins encoded by *Mx1* and *Mx2*. Other overexpressed genes included *Tlr7* and *Pdcd1* (programmed cell death protein 1 [PD-1]); we confirmed the upregulation of *Tlr7* and *Irf7* in both B cell subsets by qPCR (Fig. 6e). Three DEGs were upregulated in the CD19^+^CD138^–^ gene set but not in that from CD19^+^CD138^+^ cells (FDR>0.1), including *Alcam* (also identified above) and the early cell cycle gene *Cdk6* (Fig. 6f). Specific to CD19^+^CD138^+^ B cells was upregulation of the viral response genes *Lcn2* and *Mfsd6*, the latter of which is involved in MHC I antigen presentation (Fig. 6g).

### Pancreatic-localised B cells express Toll-like receptor 7

**Fig. 7:**
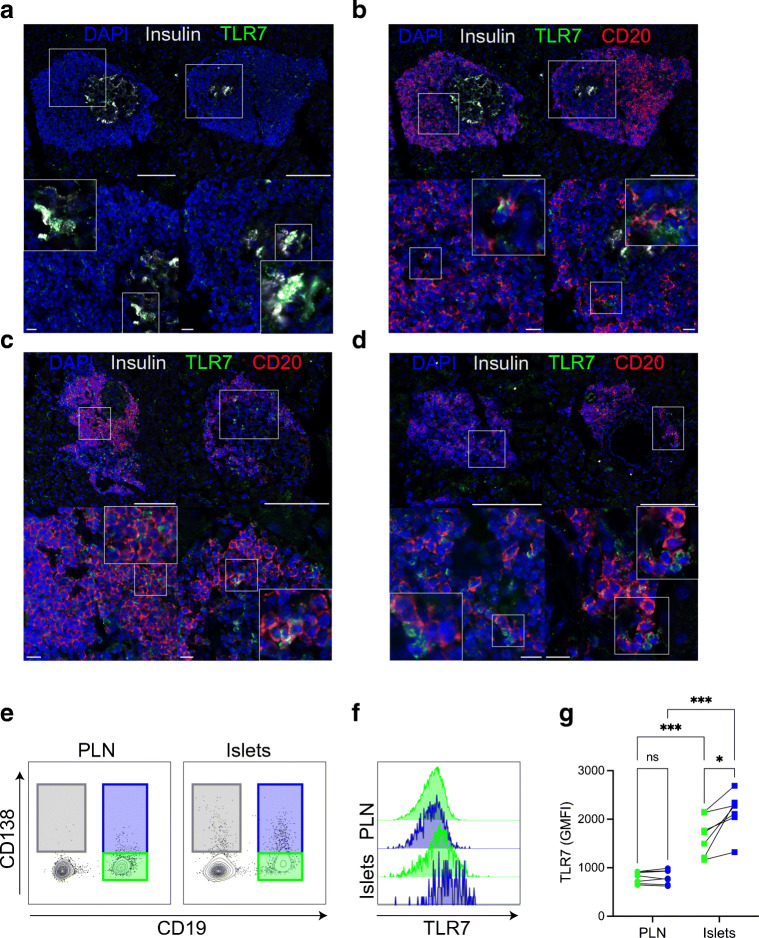
Expression of TLR7 protein in pancreatic islets in NOD mice. (**a**–**d**) Representative confocal microscopy images of pancreatic sections from female NOD mice (blood glucose >13.9 mmol/l) stained with anti-insulin (grey), anti-CD20 (red), anti-TLR7 (green) antibodies and nuclear DAPI counterstain showing (**a**) insulin-expressing beta cells positive for TLR7; (**b**) CD20^+^TLR7^+^ B cells in the remaining insulin-containing islets; (**c**) CD20^+^TLR7^+^ B cells in insulin-deficient islets; and (**d**) CD20^+^TLR7^+^ B cells in close contact with single-positive TLR7^+^ cells. Scale bars, 100 μm (top panels) and 10 μm (bottom panels). Images represent pancreatic islets or immune cell clusters from three individual mice. (**e**–**g**) PLNs and pancreatic islets from female NOD mice aged 16–20 weeks were isolated and cells analysed by flow cytometry, gated on (**e**) CD19^+^CD138^–^ (green gate), CD19^+^CD138^+^ (blue gate) and CD19^–^CD138^+^ (grey gate) B cells. (**f**) Histogram showing TLR7 expression in CD19^+^ B cell subsets. (**g**) Summary graph of TLR7 (geometric mean fluorescent intensity [GMFI]) expression. Each dot represents one individual mouse over two independent experiments. ***p<0.001 from two-way ANOVA with Bonferroni’s multiple comparison test. ns, not significant

Toll-like receptor 7 (TLR7)-deficient mice are protected from autoimmune diabetes [36] and, in individuals with systemic lupus erythematosus, TLR7 can drive autoreactive naive B cells to differentiate into pathogenic plasma cell precursors [37]. Here, we observed relatively high *Tlr7* gene expression in B cells (Fig. 6, heatmap) localised to the pancreas and hypothesised that this would be a detectable protein. We confirmed the expression of the TLR7 protein in the pancreas of NOD mice, in insulin-expressing beta cells in the remaining insulin-containing islets (Fig. 7a) and in many B cells with abundant CD20 expression (Fig. 7b). Double-positive (CD20^+^TLR7^+^) B cells were also observed in or around insulin-deficient islets (Fig. 7c). Furthermore, in the remaining islet structures and immune cell clusters, we observed both CD20^+^TLR7^+^ cells and CD20^–^TLR7^+^ cells in proximity (Fig. 7d). We demonstrated TLR7 expression in islet B cells using flow cytometry (Fig. 7e–g), enabling demarcation of our B cell subsets in PLNs and pancreatic islets (Fig. 7e). Expression of TLR7 was significantly increased in both islet CD19^+^ B cell subsets compared with their PLN counterparts, corroborating our gene expression observations (Fig. 7f, g). Of note, CD19^–^CD138^+^ cells (grey gate) showed little TLR7 expression (data not shown). However, we observed a higher level of expression of TLR7 in CD19^+^CD138^+^ B cells than in the CD19^+^CD138^–^ subset in pancreatic islets, which was not observed in PLNs (Fig. 7g).

## Discussion

We show that, in NOD mice with established insulitis, the gene expression profiles of B cells are dramatically influenced by the pancreatic environment, with inflammation favouring the upregulation of IFN-associated genes and the induction of genes promoting plasma cell differentiation. Gene expression analysis revealed a clear distinction between the populations of B cells found in PLNs and those infiltrating islets during disease development. Activated pathways were associated with innate immune signalling and included the upregulation of *Irf7* and *Tlr7* alongside proinflammatory cytokines including *Il6*, *Il1b* and *Ccl4*. Interestingly, repressed genes were associated with antigen presentation and activation, suggesting that B cells localised in the pancreas may primarily facilitate local beta cell damage by producing proinflammatory cytokines.

Our approach also highlighted a population of CD138^int^ cells enriched in pancreatic islets (compared with secondary lymphoid organs) of NOD mice, which we have previously described as a heterogeneous population of plasmablast or plasma-like cells, with downregulated CD19 and IgD but encompassing a population of insulin-specific B cells [11]. These CD138^+^ cells remain in the minority and are not selectively expanded in the pancreas during diabetes development [13] (data not shown). However, our data suggest that further expression markers, in addition to CD19 and CD138, are required to identify the bona fide B cells or plasma cells in this CD19^–^CD138^+^ pancreatic population, as we observed DEGs including both key innate lymphocyte genes and plasma cell-related genes in the gene set comparison. Recent work shows that CD138 is a marker for NKT17 cells [22], suggesting that, in pancreatic islets, such cells might be present during diabetes development. In support of this, the *Il17re* gene was activated to a greater extent in the CD138^+^CD19^–^ population than in CD19^+^ B cells. Furthermore, the CD138^+^ cell population is enriched in differentiated plasma cells, as *Jchain* was highly upregulated and a small number of IgA^+^ plasma cells were detected. Notably, in other autoimmune diseases such as multiple sclerosis, IgA^+^ B cells are enriched in inflammatory lesions in the CNS in both humans [38] and mice [39], with a role in attenuating disease via IL-10 production. The roles, significance and origin of IgA^+^ plasma cells in the pancreas are currently unknown and require further investigation.

Comparing the gene expression profile of CD19^+^ B cells in the pancreas with equivalent cells in PLNs revealed the induction of both IFN- and plasma cell-related genes. Blimp-1 and IFN regulatory factor 4 (IRF4) are key transcription factors regulating plasma cell differentiation [28, 40] and the expression of Blimp-1 leads to repression of the B cell commitment gene *Pax5*, among others [32]. Blimp-1 directly targets the MHC II regulating gene *Ciita* [30], which we show is highly repressed in B cells that have migrated to pancreatic islets. Repression of *Pax5* is required for the initiation of ASC development [41] and it is repressed in B cells present in the pancreas (FDR<0.05, >1.5-fold change). However, no difference was seen in the expression of the transcription factor XBP-1, a target of Pax-5 that acts downstream of Blimp-1 to regulate the unfolded protein response [29, 42], a process essential for the secretion of immunoglobulins. These results imply that CD19^+^ B cells located in the pancreas upregulate genes associated with antibody secretion but do not secrete immunoglobulins. Based only on gene expression profiles, we cannot discern whether pancreatic CD19^+^ B cells are precursors or pre-plasmablasts, as many genes in this pathway are transitional or are expressed continuously [26]. Functional assays and assessment of protein expression using CD19^+^ B cells from both lymph nodes and pancreas may provide more definitive evidence.

Blimp-1 and IRF4 can be activated by factors other than B cell receptor signalling, such as IL-21 and IL-6 [43] or IFNα and IL-6 [44], cytokines implicated in the pathogenesis of type 1 diabetes. IL-21 can directly induce *Prdm1* gene expression, requiring both signal transducer and activator of transcription 3 (STAT3) and IRF4 [45], which were both induced in islet B cells in this study. IFNα and IL-6, produced by plasmacytoid dendritic cells (pDCs), can induce CD40-activated B cells to differentiate into ASCs [44]. IFNα-producing pDCs in the pancreatic islets during the early stages of autoimmune diabetes are crucial for initiation of disease [7] and we demonstrate that B cells are influenced by, and acquire, this innate signature during the establishment of insulitis.

IFNα is expressed by the beta cells of patients with type 1 diabetes [46] and IFN-associated genes are overexpressed in islets of newly diagnosed individuals [47], implying a major role in the pathogenesis of type 1 diabetes. Type 1 IFNs enhance the expression of, and response to, TLR7 [48, 49], which we find highly expressed in the B cells found in pancreatic islets of NOD mice. Furthermore, in combination with IFNα, TLR7 activation augments IL-6 production and isotype switching in B cells [48]. In NOD mice, TLR7 deficiency delays and reduces the development of autoimmune diabetes and alters the functional responses of B cells [36]. Therefore, it is likely that the heightened expression of IFNα enhances TLR7 expression in pancreatic B cells and that this synergistically amplifies IL-6 and proinflammatory cytokine production. It is also important to note that TLR7-driven CD11c^+^ B cells are enhanced in autoimmune-prone mice [50] and enriched in individuals with autoimmunity [37]. The status of TLR7 in those CD20^+^ B cells in the pancreatic islets of individuals with type 1 diabetes is currently unknown, but, given that an elevated proportion of infiltrating B cells correlates with earlier diagnosis and more rapidly progressive disease [6], targeting of TLR7 could be of interest therapeutically.

Of particular importance is the heightened expression of the TLR7 protein in CD19^+^CD138^+^ islet B cells, which encompass a substantial proportion of the B cell population in the pancreas during the development and onset of diabetes in NOD mice. Furthermore, a substantial number of CD138^int^ B cells are autoreactive [11, 51]. Understanding the role of both CD138^–/+^ B cells and their relationship in the pancreatic tissue may have important consequences for B cell-targeted immunotherapy. Our gene array data revealed little difference in the CD19^+^ B cell subsets once localised within the tissue (either lymph nodes or pancreas), as each subset displayed a similar gene expression profile. However, distinct signatures were found when comparing the equivalent B cell subsets in the PLNs and pancreas, notably in the expression of selected genes such as *Igf2r*. Further studies are required to fully understand if CD138^+^ B cells fulfil a specific functional role or if they are more responsive to TLR7 ligands. It is also worth considering that the two subsets of CD19^+^ B cells are at different stages of differentiation or activation when harvested from the tissue, particularly as CD138 (syndecan-1) can be shed from the cell surface after ligand engagement [52].

Unbiased single-cell RNA sequencing studies of the pancreas of NOD mice during diabetes development [53], together with application of the approach we have employed, may shed further light on the heterogeneity among these specific B cell subsets. A further limitation of the present study is that we do not address B cell specificity, which, if understood more fully, may help to highlight key differences in functionality of the CD138^+^ B cell subsets. Despite this, our observations provide new and important findings indicating how B cells may contribute to local beta cell damage and perpetuate tissue inflammation. Taken together, our study provides novel insights into potential therapeutic avenues that may be effective in individuals with type 1 diabetes.

## Supplementary information

ESM (PDF 1103 kb)

ESMTables 2–5 (XLSX 416 kb)

## Data Availability

The datasets generated and/or analysed during the current study are available from the corresponding author on reasonable request.

## Abbreviations

ASC: Antibody-secreting cell
Blimp-1: B lymphocyte-induced maturation protein 1
DEG: Differentially expressed gene
FDR: False discovery rate
GO: Gene ontology
IRF4: IFN regulatory factor 4
IPA: Ingenuity pathway analysis
PLN: Pancreatic lymph node
qPCR: Quantitative PCR
TLR7: Toll-like receptor 7
XBP-1: X-box binding protein 1
