# Supplementary material for: Gene expression profiling in NOD mice reveals that B cells are highly educated by the pancreatic environment during autoimmune diabetes
Source: Diabetologia. 2022 Dec 12;66(3):551–66. doi: 10.1007/s00125-022-05839-7 (PMC9892163; doi:10.1007/s00125-022-05839-7)
Supplement: Supplementary file 1 — (PDF 1103 kb) [file 125_2022_5839_MOESM1_ESM.pdf]

Supplementary Methods

Tissue preparation Pancreatic lymph nodes (PLNs) were disrupted mechanically with a 30G needle. Pancreata were inflated with collagenase P solution (1.1 mg/mL) (Roche, Welwyn Garden City, U.K.) in Hanks' balanced salt solution (with Ca2 and Mg2) through the common bile duct, followed by collagenase digestion with shaking at 37°C for 10 min. Islets were isolated by Histopaque density centrifugation (Sigma-Aldrich, Dorset, U.K.) and hand-picked under a dissecting microscope. For flow cytometric sorting, islets were then trypsinised to generate a single-cell suspension. Islet cells were rested at 37°C in 5% CO2 in Iscove' s modified Dulbecco's media (supplemented with 5% FBS, 2 mmol/L L-glutamine, 100 units/mL penicillin, 100 m g/mL streptomycin, and 50 mmol/L β-2-mercaptoethanol) overnight.

Flow Cytometry and Fluorescent Activated Cell Sorting (FACS)

B-cell phenotyping multiparameter flow cytometry was performed using the following mAbs: CD19 (6D5), CD138 (281-2), B220 (RA3-6B2), IgD (11-26c.2a), IgM (RMM-1), CD3 (145-2C11), CD11c (N418), CD11b (M1/70), CD127 (A7R34). For TLR7 (A94B10, BioLegend) staining, cells were fixed/permeabilized using eBioscience nuclear transcription kit. FACS was carried out with the following mAbs: CD3 (145-2C11), CD11c (N418), CD11b (M1/70), CD19 (6D5), CD138 (281-2), IgD (11-26c.2a). Dead cells were excluded using live/dead exclusion dye (Invitrogen).

Immunofluorescence Pancreatic tissues were frozen in optimal cutting temperature (OCT) medium and sectioned at 7-10μm thickness. Pancreatic sections were fixed in 1% paraformaldehyde for 1 h at room temperature. Following fixation, tissue was permeabilized with 0.2% Triton X-100 and blocked with 5% FBS before the addition of a primary antibody mix before secondary labelling was performed. Primary antibodies used were rat or rabbit anti-mouse CD20 (Abcam [1/50 dilution]; Cell Signalling [1/100 dilution] respectively), rat anti-

mouse CD138 ([1/100] Clone 281.2, BioLegend), directly conjugated goat anti-mouse IgA ([1/200] Cambridge Bioscience), rabbit anti-mouse TLR7 ([1/100] Novus Biologicals), biotinylated anti-insulin ([1/200] clone D6C4; Abcam). Secondary antibodies used were Alexa Fluor 488 ([1/200] Invitrogen), Alexa Fluor 647 ([1/100] Abcam), Streptavidin-conjugated Alexa Fluor 568 ([1/100] Invitrogen). Antibodies were validated on splenic tissue. All slides were mounted with VECTASHIELD mounting medium with DAPI (Vector Laboratories). All sections were imaged on a Leica DMi8 TCS SP8 Confocal 8 and processed in Fiji Image J (Version 2.0).

RNA isolation RNA was isolated from cells with Qiagen RNAeasy Micro kit, according to manufacturer's instructions. B cell subsets were sorted directly into RLT buffer (Qiagen) and RNA isolated immediately. Total RNA was quantified, and quality assessed using the RNA 6000 Pico Kit on the Agilent 2100 Bioanalyzer (Agilent Technologies, Palo Alto, CA, USA).

Clariom S array RNA amplification was achieved using low-cycle PCR followed by linear amplification using T7 *in vitro* transcription (IVT) technology. For each sample, a hybridization cocktail of biotinylated target was incubated with a GeneChip® Mouse Clariom S array (ThermoFisher Scientific) at 60rpm for 16hours at 45°C in a Genechip® Hybridisation Oven 645 (Affymetrix). After hybridization, non-specifically bound material was removed by washing and specifically bound target was detected using the GeneChip® Hybridization, Wash and Stain Kit (ThermoFisher Scientific), in conjunction with the GeneChip® Fluidics Station 450 (Affymetrix). The arrays were scanned using a GeneChip® Scanner 3000 7G (Affymetrix) in conjunction with Affymetrix Genechip® Command Console (AGCC) software and .CEL files were generated from the resultant probe cell intensity data.

Gene array analyses Genes were annotated using the Affymetrix 'clariomsmousetranscriptcluster.db' annotation data package (Bioconductor). For further analysis the ebayes method was used (TAC software) to compare gene expression with

previously published data and perform Venn analysis. The PANTHER classification system [1] or the Functional Annotation Tool DAVID [2] was used for gene ontology (GO) analysis with either upregulated or downregulated genes (FDR<0.05, FC >2). The statistical overrepresentation analysis tool was used in PANTHER to determine significant GO terms. Significant gene regulatory networks and pathways were investigated using Ingenuity Pathway Analysis (Qiagen Bioinformatics) and quantified with a Z-score and -log(*p*-value). All genes were uploaded and an FDR<0.05 and a fold change >1.5 threshold set. Finally, bubble plots were generated using ggplot2 Rstudio package (version 1.2.5042), hierarchical clustering heatmaps were generated in Rstudio with either ggplot2 or Pheatmap packages. Other heatmaps, volcano or scatter plots and principal component analysis (PCA) were performed in GraphPad Prism v9.

[1] Mi H, Muruganujan A, Casagrande JT, Thomas PD (2013) Large-scale gene function analysis with the PANTHER classification system. Nat Protoc 8(8): 1551-1566. 10.1038/nprot.2013.092

[2] Huang da W, Sherman BT, Lempicki RA (2009) Systematic and integrative analysis of large gene lists using DAVID bioinformatics resources. Nat Protoc 4(1): 44-57. 10.1038/nprot.2008.211

**B cells in the pancreas**	**Phenotype**	**Insulitis stage**
$CD19^{+}CD138^{-}$	$CD5^{-}$, IgM^{lo}, IgD^{+}	Early > Established
$CD19^{+}CD138^{+}$	$CD5^{-}$, IgM^{lo}, IgD^{+}, Autoreactivity	Early >> Established
$CD19^{+}CD138^{hi}$ (plasmablast)	$CD5^{-}$, IgM^{lo}, IgD-, $CD44^{hi}$, $Ki67^{+}$	Early > Established
$CD19^{-}CD138^{+}$	IgD^{-}, Bone fide B cell?	Early - Established
B1a	$CD5^{+}$	Early < Established

ESM Table 1. B cells identified to be in the pancreas of NOD mice during the development of autoimmune diabetes.

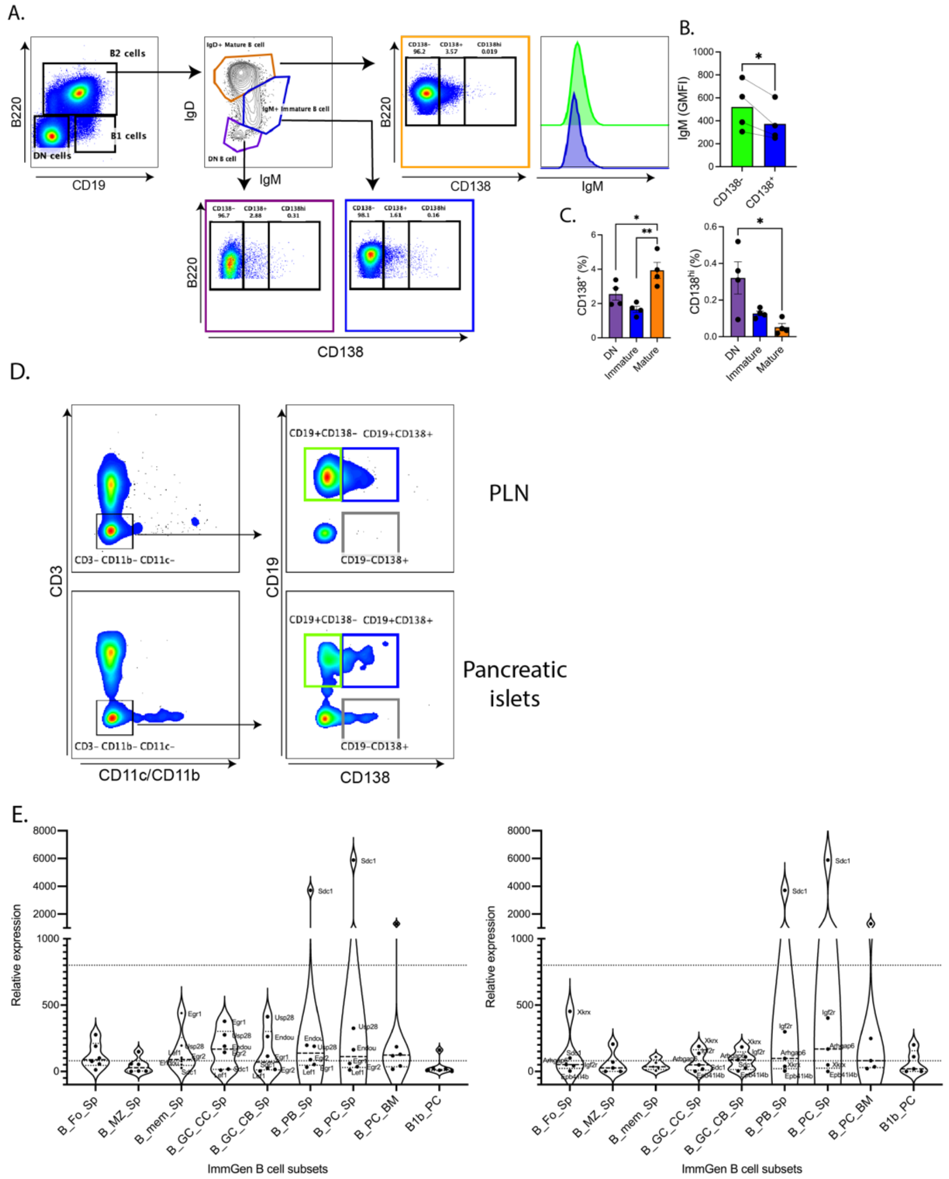

ESM Fig. 1 Comparison of CD19$^+$CD138$^-$ and CD19$^+$CD138$^+$ B cells. (A-C) B cells from NOD splenocytes were stained for B cell markers to show CD138$^+$ B cells are enriched in the mature B cell population (A) representative flow cytometry plots (B) IgM GMFI (mean fluorescence intensity) on both CD138$^-$ and CD138$^+$ populations, *<0.05, paired t-test (C) Histograms to show CD138$^+$ (left) and CD138^{hi} (right) percentages in the DN (double negative), immature and mature B cell subsets. *<0.05, **<0.01, One-way ANOVA. (D) Gating strategy for FACS sorting B cells in the PLN (top) and pancreatic islets (bottom) (E) Violin plots for the relative gene expression acquired from the ImmGen database for distinct B cell populations. Dots represent relative gene expression from selected/chosen genes related to Figure 2A in each ImmGen subset. Selected genes are related to expression differences between CD19$^+$CD138$^-$ and CD19$^+$CD138$^+$ B cells in the PLN (left) and the pancreatic islets (right) shown in Figure 2A. B cell populations (derived from ImmGen) described on the x-axis are B_Fo_Sp (Follicular zone B cells, spleen), B_MZ_Sp (Marginal zone B cells, spleen), B_mem_Sp (Memory B cells), B_GC_CC_Sp (Germinal centre centrocyte B cells, spleen), B_GC_CB_Sp (Germinal centre centroblast B cells), B_PB_sp (Plasmablast, spleen),

B_PC_Sp (Plasma cell, spleen), B_PC_BM (Plasma cell, bone marrow), B1b_PC (B1b plasma cell).

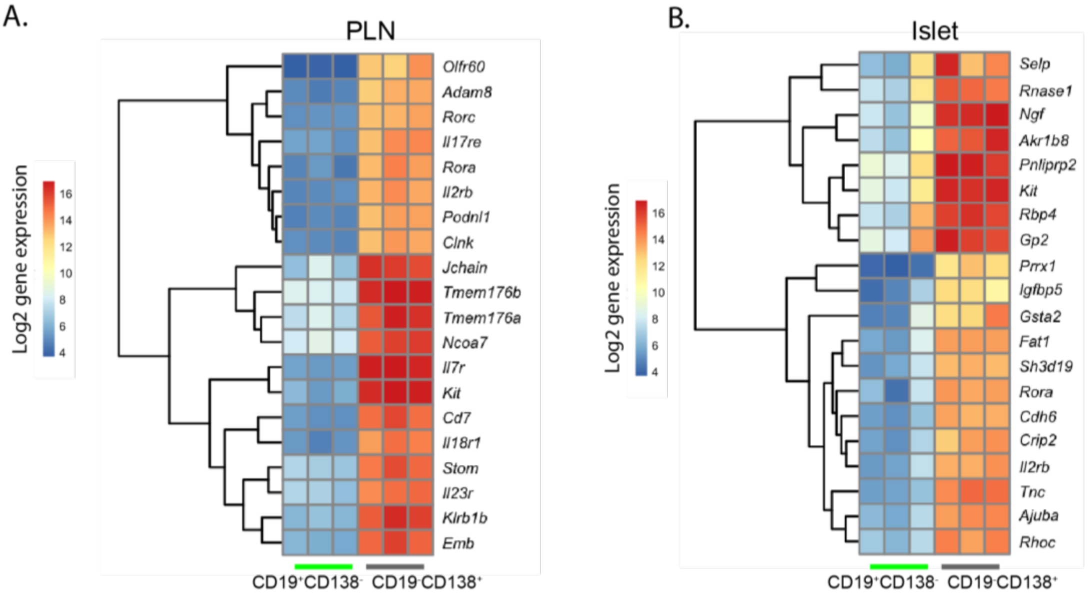

ESM Fig 2 The CD19^{-}CD138^{+} subset contain innate lymphocyte population. Gene expression related to Figure 2. (A, B) Hierarchical clustering heatmaps to show the most significant (FDR *p*<0.05, >2-fold) change genes differentially expressed when comparing CD19^{+}CD138^{-} B cells to CD19^{-}CD138^{+} subsets in both (A) PLN and (B) pancreatic islets.

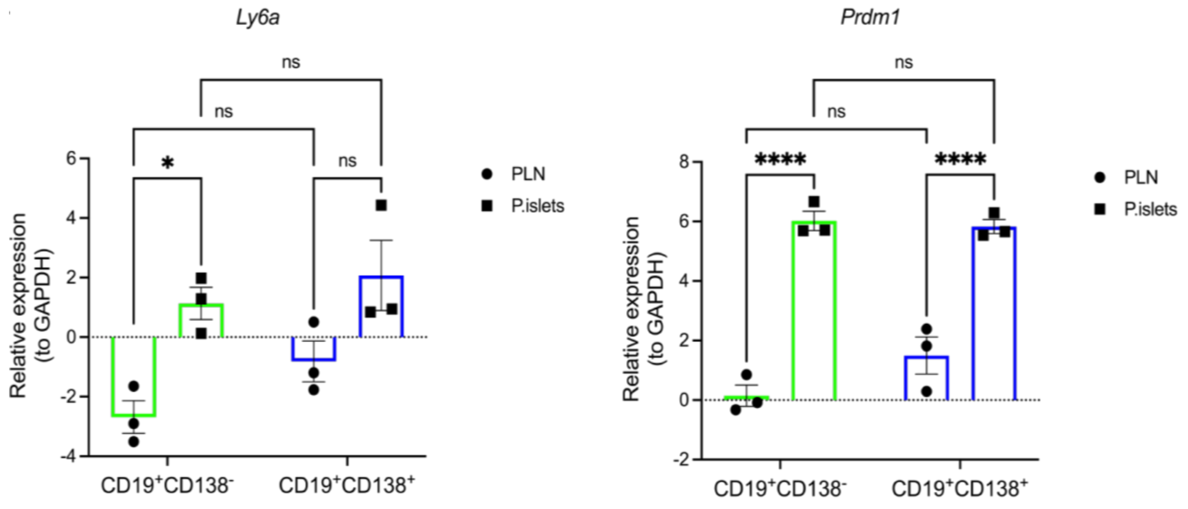

ESM Fig 3 Pancreatic islet-specific B cells upregulate antibody secreting genes. Related to Figure 4. qPCR of *Ly6a* and *Prdm1* genes in B cell subsets located in the PLN and pancreatic islets. Expression was related to housekeeping gene GAPDH. *<0.05, ****<0.0001, ns: not significant, two-way ANOVA with a Bonferroni's multiple comparison test.

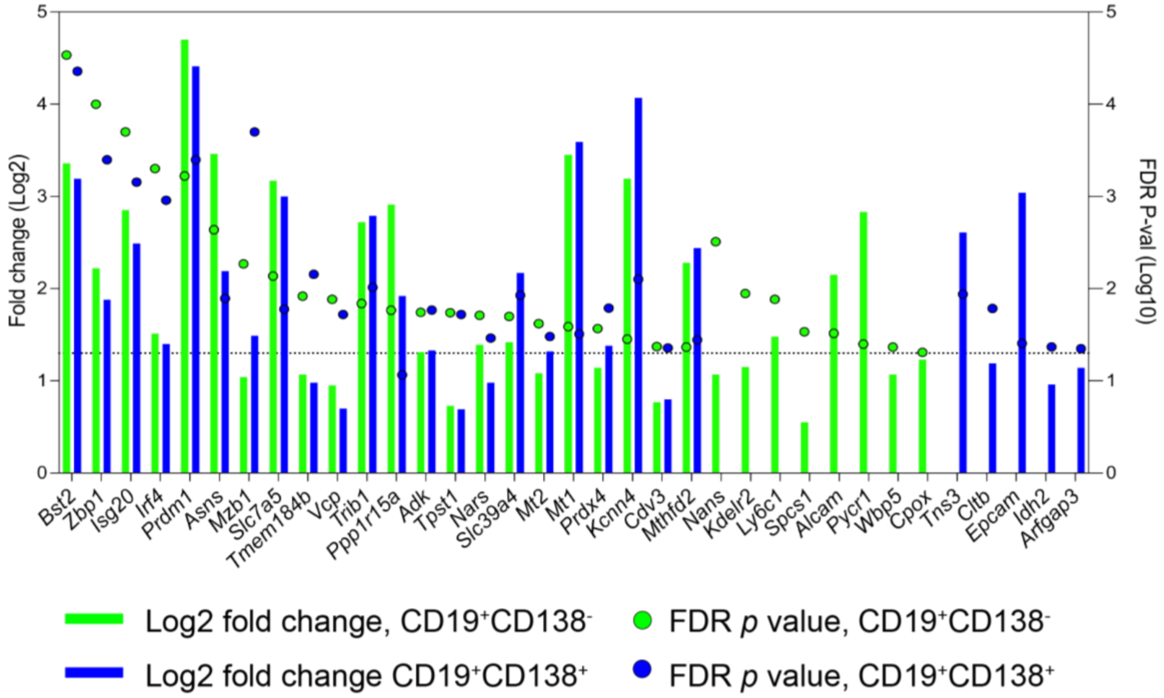

ESM Fig 4 Islet-specific B cells are enriched in genes associated with antibody secreting cells. Bar graphs showing Log2 fold change (left y-axis) and FDR *p*-value (right y-axis) for $CD19^{+}CD138^{-}$ (green bars) and $CD19^{+}CD138^{+}$ (blue bars) B cell gene sets (PLN vs pancreatic islets). Dotted line indicates FDR *p*-value 0.05. Genes not expressed in B cell subsets (single bars) had an FDR *p*-value>0.1.

response to virus (GO:0009615)
response to external biotic stimulus (GO:0043207)
defense response to symbiont (GO:0140546)
response to other organism (GO:0051707)
defense response to virus (GO:0051607)
response to stress (GO:0006950)
defense response to other organism (GO:0098542)
response to biotic stimulus (GO:0009607)
biological process involved in interspecies interaction between organisms (GO:0044419)
response to external stimulus (GO:0009605)
defense response (GO:0006952)
innate immune response (GO:0045087)
immune system process (GO:0002376)
immune response (GO:0006955)
response to interferon-beta (GO:0035456)
response to cytokine (GO:0034097)
cellular metabolic process (GO:0044237)
organic substance biosynthetic process (GO:1901576)
response to interferon-alpha (GO:0035455)
negative regulation of biological process (GO:0048519)
regulation of cell death (GO:0010941)
regulation of apoptotic process (GO:0042981)
regulation of programmed cell death (GO:0043067)
cellular nitrogen compound metabolic process (GO:0034641)

ESM Fig 5 List of Gene Ontology (GO) terms. Related to Figure 5.
